# Ex Vivo Organotypic Brain Slice Models for Glioblastoma: A Systematic Review

**DOI:** 10.3390/cancers18030372

**Published:** 2026-01-25

**Authors:** Cateno C. T. Petralia, Agata G. D’amico, Velia D’Agata, Giuseppe Broggi, Giuseppe M. V. Barbagallo

**Affiliations:** 1 Department of Neurosurgery, Policlinico “G. Rodolico—San Marco” University Hospital, 95123 Catania, Italy; giuseppebarbagal@hotmail.com; 2 Department of Drug and Health Sciences, University of Catania, 95124 Catania, Italy; agata.damico@unict.it; 3 Section of Anatomy, Histology and Movement Sciences, Department of Biomedical and Biotechnological Sciences, University of Catania, 95124 Catania, Italy; vdagata@unict.it; 4 Department of Medical and Surgical Sciences and Advanced Technologies “G.F. Ingrassia” Anatomic Pathology, University of Catania, 95124 Catania, Italy; giuseppe.broggi@gmail.com

**Keywords:** glioblastoma, ex vivo models, organotypic brain slice cultures, tumour microenvironment, invasion, drug testing, translational research

## Abstract

Background/Objective: This systematic review aims to evaluate ex vivo brain slice models in glioblastoma (GBM) research, with a specific focus on tumour invasion, tumour–microenvironment interactions, and therapeutic response. Methods: A systematic search looking for studies employing ex vivo organotypic brain slice models in GBM research was conducted across multiple databases (January 2010–July 2025) in accordance with PRISMA guidelines. The study was registered in PROSPERO database (CRD420251138341). Inclusion criteria encompassed patient-derived brain slices, hybrid rodent–human slice co-cultures, and microfluidic-integrated ex vivo platforms designed to assess tumour invasion, microenvironmental interactions and therapeutic responses. Exclusion criteria included reviews, abstracts, conference proceedings, in vivo-only studies, purely in vitro models without organotypic integration, and studies not focused on GBM. Results: Twenty-six studies met the inclusion criteria. Among these, 18/26 (69%) investigated GBM invasion, 18/26 (69%) evaluated therapeutic responses, and 5/26 (19%) examined tumour–microenvironment interactions, with several studies spanning multiple domains. Across platforms, organotypic slices consistently recapitulated key features of GBM biology—including perivascular and white-matter-aligned invasion, stromal–immune interactions, and patient-specific drug sensitivity—while engineered systems enhanced perfusion and exposure control. Methodological variability, particularly regarding slice preparation, oxygenation and viability assessment, limits direct comparability between studies. Conclusions: Organotypic brain slice models represent an extremely relevant tool for translational investigations of GBM biology and treatment response. However, substantial methodological heterogeneity together with limited standardisation hamper reproducibility and cross-study validation. Future work should focus on enhancing reproducibility and harmonising protocols to support the development of clinically meaningful precision oncology strategies.

## Simple Summary

Glioblastoma is considered the most aggressive primary brain tumour and despite advances in surgery, radiotherapy and chemotherapy prognosis remains dire. Preclinical models play a fundamental role in understanding glioblastoma biology and testing novel therapeutic strategies, however both in vitro and in vivo models present important limitations. Ex vivo organotypic brain slice cultures allow the preservation of native brain structure and key microenvironmental features, providing a valuable intermediate platform between cell cultures and animal models. In this systematic review, we summarise and critically analyse the application of ex vivo organotypic brain slice models in glioblastoma research. Moreover, we highlight their main experimental applications, methodological heterogeneity and intrinsic limitations, with particular attention to translational relevance and standardisation challenges. This work aims to provide researchers with a clear framework to understand existing studies and to support the rational design of future ex vivo glioblastoma models.

## 1. Introduction

Glioblastoma (GBM) is the most common and aggressive primary malignant brain tumour in adults [1]. Despite maximal safe resection followed by radiotherapy and adjuvant chemotherapy (Stupp protocol), median overall survival remains approximately 14–18 months [2,3]. Even with the addition of new strategies such as tumour-treating fields [4] or vaccination against known relevant mutations, most patients experience recurrence within one year after surgical resection, and the five-year survival rate remains below 10% [5]. Among the accepted reasons for this dismal prognosis is marked intratumoural heterogeneity combined with high therapy resistance and a highly infiltrative growth pattern, making complete surgical resection virtually impossible with current technology [6].

The traditional approach for studying GBM has relied on two-dimensional cell cultures, which allow rapid manipulation [7,8,9]. However, this technique fails to replicate the so-called tumour microenvironment (TME), including the extracellular matrix (ECM), stromal cells surrounding the tumour, blood vessels and the soluble factors such as growth factors and cytokines [10,11,12]. In vivo xenograft models are considered excellent for preserving tumour heterogeneity and invasion patterns; however, they are costly, time-consuming and limited by interspecies differences that alter immune and stromal interactions [13]. Over the past two decades, spheroids and organoids have gained popularity as three-dimensional in vitro models, providing insights into tumour–cell organisation and drug response [14]. However, these systems lack the surrounding cytoarchitectural context present in vivo, which plays an important role in modulating tumour and healthy brain cell behaviour. Taken together, these experimental platforms reflect a balance between experimental simplicity, biological fidelity and translational relevance, with organotypic brain slice cultures able to bridge the gap between simplified in vitro and complex in vivo models.

Described for the first time by Stoppini and colleagues to study hippocampal physiology [15], brain slices involve ex vivo culture of thin brain sections that retain native cytoarchitecture, vasculature, extracellular matrix and resident non-neuronal cells [16,17]. Because of these properties, ex vivo organotypic brain slices have been increasingly employed to explore invasion patterns, tumour–stroma interactions and therapeutic responses.

Since Stoppini’s model, several distinct variants of brain slice culture models have been developed. The classical model typically employs rodent slices implanted with human glioma cells or spheroids [18]. More recently, patient-derived slices have emerged as the preferred protocol due to improved preservation of human cytoarchitecture [19]. Finally, hybrid systems have also emerged, including xenograft-derived slice cultures and microfluidic “slice-on-a-chip” platforms. Xenograft-based slices are generated from orthotopic tumours [20], allowing investigators to study GBM cells within a perfused and structurally intact environment. Microfluidic “slice-on-a-chip” systems [21] introduce greater control over perfusion, nutrient flow and waste removal, extending slice viability and enabling multiplexed drug testing. To provide a general conceptual framework, the main variants of organotypic brain slice culture models developed since the original Stoppini’s protocol, together with their main applications, advantages, and limitations, are summarised in Table 1.

The applications of organotypic brain slices in GBM research are diverse, reflecting their ability to preserve key elements of the TME. These models are particularly useful in elucidating mechanisms of tumour cell proliferation and therapy resistance. Furthermore, other studies have used these platforms to explore tumour–immune interactions, such as microglial activation, T-cell infiltration and macrophage migration [19,22]. In addition, several studies have investigated therapeutic agents, including kinase and histone deacetylase (HDAC) inhibitors, radiosensitisers, and targeted compounds with potential clinical relevance [23]. More recently, integrating microfluidic chemosensitivity assays and high-resolution imaging has expanded the translational potential of slice cultures, enabling dynamic assessment of treatment response under near-physiological conditions [21].

Despite increasing adoption, significant heterogeneity exists among slice culture methodologies. Protocols vary in host species (mouse, rat, or human), slicing technique (vibratome vs. tissue chopper), thickness (200–400 µm), culture duration (short vs. extended), implantation strategy (cell injection, tumour-spheroid placement, or native tumour slice), and experimental endpoints (invasion distance, proliferation indices, transcriptomic profiling). This substantial variability makes rigorous cross-study comparison difficult and limits the establishment of standardised practices [19,24]. Furthermore, although previous studies have extensively explored experimental models used in glioblastoma research, including two-dimensional cultures, three-dimensional systems, and in vivo approaches [7,9,13,14], no comprehensive and systematic synthesis has specifically evaluated ex vivo organotypic brain slice models across tissue sources, methodological parameters and experimental applications.

Therefore, this systematic review aims to critically evaluate the application of ex vivo organotypic brain slice models in GBM research. Specifically, we examine methodological heterogeneity, tumour invasion, treatment response and translational relevance of these models compared with other commonly used preclinical systems. Through this systematic review, our goal is to consolidate current knowledge, inform future experimental design and highlight the role of these models in bridging conventional in vitro assays and clinically oriented translation research.

**Table 1:** Overview of the main variants of organotypic brain slice culture models developed since the original Stoppini protocol. This table provides a conceptual comparison of the principal organotypic brain slice platforms used in neuro-oncology research, summarizing tissue sources, main experimental applications, key advantages, and limitations. Classification is based on widely adopted methodological descriptions in the literature [15,16,17,19,22,25].

Model Variant	Tissue Source	Main Experimental Application	Main Advantages	Main Limitations
Rodent-derived organotypic brain slices	Mouse or rat brain (postnatal or adult)	Mechanistic studies of tumour invasion and migration	High reproducibility, wide availability, good imaging accessibility, standardised protocols	Species mismatch, limited human immune and stromal relevance
Human patient-derived tumour slices	Surgical GBM tissue	Patient-specific invasion profiling and therapeutic response assessment	Preservation of native human cytoarchitecture and tumour heterogeneity	Limited availability, inter-patient variability, lower scalability
Human nontumoural brain slices with GBM cell implantation	Human cortical tissue (e.g., epilepsy surgery)	Controlled tumour–host interaction and invasion modelling	Human extracellular matrix and vasculature preserved, controlled tumour seeding	Lacks native tumour architecture and endogenous tumour microenvironment
Xenograft-derived organotypic slices	Orthotopic human GBM xenografts in rodents	Bridging in vivo and ex vivo tumour behaviour	Maintains tumour structure and perfusion-related features	Mixed-species environment, altered immune context
Hybrid/engineered organotypic systems (e.g., tandem cultures, slice-on-a-chip)	Human and/or rodent slices with microengineering	Drug screening, perfusion-controlled assays, precision oncology applications	Improved control of nutrient/drug delivery, extended viability, multiplex testing	Technical complexity, lower throughput, limited standardisation

## 2. Methods

This systematic review was conducted according to the PRISMA 2020 guidelines and the review protocol was prospectively registered in PROSPERO (CRD420251138341).

### 2.1. Search Strategy

We conducted a comprehensive systematic literature review search in PubMed, Embase (Ovid), Web of Science and Scopus for studies published from January 2010 to July 2025. In addition, Google Scholar was searched as a supplementary source to identify further potentially relevant studies not elsewhere indexed. This supplementary search did not yield additional eligible studies beyond those identified through the primary database search. The final search update was performed in July 2025; no additional eligible studies published in 2025 meeting the inclusion criteria were identified. The starting year of 2010 was selected to focus on studies reflecting the contemporary application of organotypic brain slice cultures in glioblastoma research, particularly those addressing tumour invasion, microenvironmental interactions, and therapeutic testing, while earlier studies predominantly focused on neurophysiology or exploratory tumour models with limited clinical relevance.

Search strategies combined database-specific controlled vocabulary (e.g., MeSH and Emtree terms) with free-text keywords related to organotypic or ex vivo brain slice cultures (“organotypic slice”, “brain slice”, “tissue slice culture*”, “ex vivo slice*”) and gliomas (“glioblastoma”, “GBM”, “glioma*”). Boolean operators and truncation were adapted for each database. We did not apply any language restrictions during the initial search to minimise selection bias. Reference lists of included studies were screened manually to identify any additional eligible articles. Database searches identified 1301 records before deduplication.

### 2.2. Inclusion and Exclusion Criteria

Studies were included if they met all of the following criteria:

- Model: preclinical experimental studies investigating ex vivo organotypic brain slice cultures.

Accepted models included the following: patient-derived GBM tissue slices, glioma cells or GBM stem-like cells implanted on rodent slices, and hybrid organotypic systems (i.e., xenograft-derived slices and microfluidic platforms);

- Focus: investigation of GBM biology, including tumour invasion and cell migration, tumour–microenvironment interactions and therapeutic response to treatment;
- Study type: peer-reviewed original research articles.

Exclusion criteria: reviews, abstracts, posters, editorials, dissertations, in vivo studies not including ex vivo slice models and in vitro models without integration with organotypic brain slices.

### 2.3. Screening and Selection

**Figure 1:**
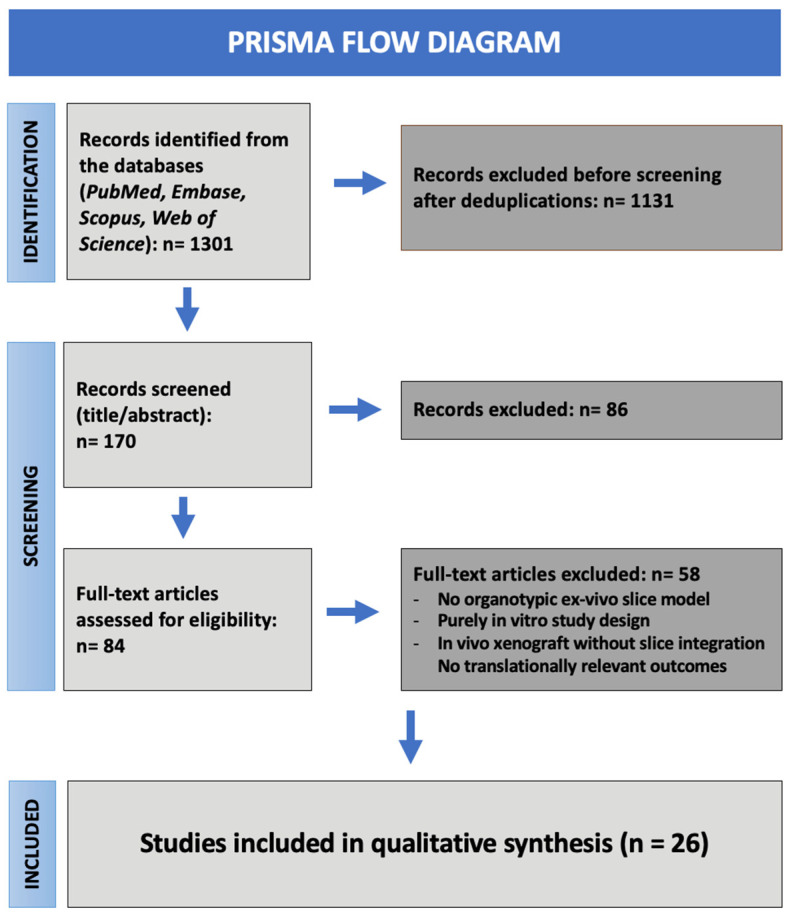
PRISMA 2020 flow diagram of study selection. The diagram summarises the identification, screening, eligibility assessment and inclusion of studies evaluating ex vivo organotypic brain slice models in glioblastoma research. Following database searching and full-text screening, 26 studies were included in the final qualitative analysis.

All records retrieved from the initial search were imported into Rayyan (Qatar Computing Research Institute) for automated deduplication and blinded screening. After deduplication, 170 unique records remained. Title and abstract screening removed 86 irrelevant articles, leaving 84 records for full-text review. Following full-text assessment, 26 studies met the inclusion criteria and were included in the final synthesis. The study selection process is illustrated in the PRISMA flowchart (Figure 1).

### 2.4. Data Extraction

Data were extracted into a standardised spreadsheet capturing the following: author and year of publication, species and tissue source, slice type, slice preparation parameters (including thickness and slicing method), tumour modelling strategy, culture duration, experimental interventions (such as pharmacological treatments or genetic manipulation), outcome measures (including invasion, proliferation, apoptosis and tumour–microenvironment interactions), and main experimental focus.

Extracted data were organised into two complementary tables. Table 2 summarises the general characteristics and classification of the ex vivo organotypic brain slice models employed across the included studies, while Table 3 provides a detailed overview of key methodological features, experimental interventions, outcome measures and reported limitations, facilitating cross-study comparison.

Results were synthesised descriptively to identify common methodological patterns and application domains across studies.

### 2.5. Risk of Bias and Quality Assessment

Since the included studies employed heterogeneous ex vivo organotypic brain slice models rather than standardised in vivo animal experiments or interventional clinical studies, the application of formal risk-of-bias tools such as SYRCLE or ROBINS-I was not appropriate. In fact, these tools are designed for in vivo or clinical study designs and do not capture model-specific sources of bias relevant to ex vivo platforms such as tissue viability, slice preparation, culture conditions and endpoint selection. Accordingly, methodological quality was assessed narratively, with attention to reporting transparency, experimental controls and translational relevance. Data extraction and appraisal were conducted independently by two of the reviewers. In cases of disagreement, a joint review of the full-text article was performed until a full consensus was achieved.

### 2.6. Data Synthesis

Due to methodological heterogeneity, meta-analysis was not feasible. Data were synthesised across four domains: (i) model diversity, (ii) TME interaction, (iii) GBM cell invasion and migration, and (iv) therapeutic testing and translational relevance. Data trends and knowledge gaps were summarised narratively, and the key variables were identified and tabulated.

## 3. Results

### 3.1. Study Selection

The initial database search yielded 1301 records across PubMed, Embase, Scopus, Web of Science and Google Scholar. After removing duplicates and conducting initial screening, 170 unique articles remained. Through title and abstract screening, we excluded 86 articles that did not meet the inclusion criteria, leaving then 84 articles for full-text review. After full-text review, 58 articles were excluded for lacking an ex vivo organotypic slice model, applying purely in vitro systems, employing in vivo xenograft without integration with brain slice cultures or failing to report translation relevant outcomes. Therefore, we identified 26 studies that were finally included in our qualitative synthesis. The study selection process is summarised in the PRISMA flow diagram (Figure 1).

### 3.2. Characteristics of Included Studies

The 26 articles included were published between 2011 and 2024. All studies were preclinical investigations employing a diverse set of ex vivo organotypic brain slice cultures to study GBM.

The main characteristics of the included studies and the classification of organotypic brain slice models are summarised in Table 2.

#### 3.2.1. Model Types and Slice Source

Across the included studies, we identified three main model categories:

- Rodent-derived slices: Fourteen studies (54%). These slices were typically obtained from postnatal day 3–14 mouse or rat brains and represented the most commonly used tissue platform. These models used rodent brain slices as a scaffold for human glioma cells (predominantly GBM-derived) or GBM stem cells (GSCs) [20,26];
- Human patient-derived slices: Ten studies (38%). In these studies, slices were generated directly from human surgical specimens [19,22,27];
- Hybrid or engineered platforms: Two studies (8%). These studies applied more complex configurations combining different tissue sources or microengineering. This group includes the tandem slice system from Sidorcenco et al. [28] and the platform designed by Mann et al. using human GBM tissue perfused on rodent host slices [25].

#### 3.2.2. Slice Thickness and Culture Duration

Across the 26 studies, thickness ranged approximately from 200 to 400 µm.

- Thin slices (~200–250 µm) are typically used in studies aiming to report high-resolution invasion and drug testing assays [20,29,30];
- Intermediate slices (250–350 µm) are the most commonly used across both rodent and human models [19,21,22];
- Thick slices (≥350–400 µm) are employed when preserving vascular niches or white-matter organisation is required [31].

The majority of the slices were prepared using a vibratome (94%).

Culture duration varied by tissue source:

- Rodent slices: generally viable for 3–10 days, sufficient for mechanistic assays [32,33];
- Human slices: typically were maintained for 7–21 days, enabling longer-term analysis of TME behaviour [19,24];
- Extended culture: up to ≥35 days under optimised conditions [34];
- Hybrid/microfluidic systems: usually viable for 7–15 days with improved perfusion [35].

### 3.3. Methodological Heterogeneity

Across the 26 studies included, we observed a significant methodological variability, particularly in slice preparation, tumour seeding strategies, culture media, oxygenation, and outcome quantification. These differences influenced cell viability, TME preservation and invasion assays. A structured summary of key methodological parameters across studies is presented in Table 3, while their detailed classification into optimal and suboptimal conditions is reported in Appendix A.

#### 3.3.1. Slice Origin Variability

- Rodent tissues showed greater methodological consistency with most studies using rodent tissue from animals between 3 and 14 days old [26,33];
- Human tissue showed greater biological variability, including slices from either GBM resections or non tumoural cortical slices from epilepsy or functional resections, used as substrate for GBM cell implantation [19,22,27].

#### 3.3.2. Functional Impact of Thickness Choices

Slice thickness selection directly affected

- white-matter preservation [31];
- oxygen/nutrient gradients [22];
- single-cell tracking resolution [20,29].

Overall, slice thickness emerges as a critical experimental parameter, as it directly modulates tissue viability, invasion dynamics and the reliability of functional results, including migration analysis and therapeutic response assessment.

#### 3.3.3. Tumour Introduction Strategies

Three major implantation approaches were identified:

1. Microinjection of dissociated tumour cells (45%)

This technique was used in a protocol aimed at quantitative invasion assays and real-time motility tracking [35].

2. Spheroid placement on slice surface (30%)

These models were used to study tumour invasion, ECM interaction and therapeutic responses [18,36].

3. Native tumour tissue without implantation (25%)

These models were employed only in human-derived slice studies since they allowed direct evaluation of patient-specific invasion and drug sensitivity [25,37].

#### 3.3.4. Culture Media, Oxygenation and Environmental Conditions

Culture media

Media composition varied significantly across the studies:

- Serum-free media such as Neurobasal or DMEM/F12 + B27/GlutaMAX) were used in ~70% of studies [22,24];
- Low-serum or serum-containing media were used for short-term assays [27];
- Specialised stem cell media were used to support GSC or stromal populations’ survival [34,38].

Oxygenation and perfusion

Few studies explicitly reported oxygenation, but when described, controlled O_2_ tension or perfusion improved viability and preserved microglia and vasculature [22,35].

#### 3.3.5. Outcome Measures and Quantification Workflows

Common endpoints included the following:

- Invasion metrics: radial distance, trajectory mapping, perivascular migration [21,26];
- Viability/proliferation: Ki-67, cleaved caspase-3, TUNEL, LDH [18,19];
- TME-related readouts: microglial activation, astrocytic reactivity, cytokines [36,39];
- Drug response: cytotoxicity, apoptosis, pathway analysis [20,38].

#### 3.3.6. Reporting Quality and Sources of Bias

Notably, although, as discussed in the Methods Section, risk-of-bias tools were not applicable, several structural limitations were noted:

- Limited reporting of viability controls (only ~20% of studies used systematic live/dead or LDH assays);
- Inconsistent documentation of oxygenation and perfusion parameters;
- Blinding and randomisation;
- Sample-size justification absent in nearly all studies;
- Media composition often reported incompletely.

Despite these limitations, most studies showed an appropriate methodological clarity throughout their protocols.

### 3.4. Applications of Organotypic Brain Slice Models in Glioblastoma Research

**Figure 2:**
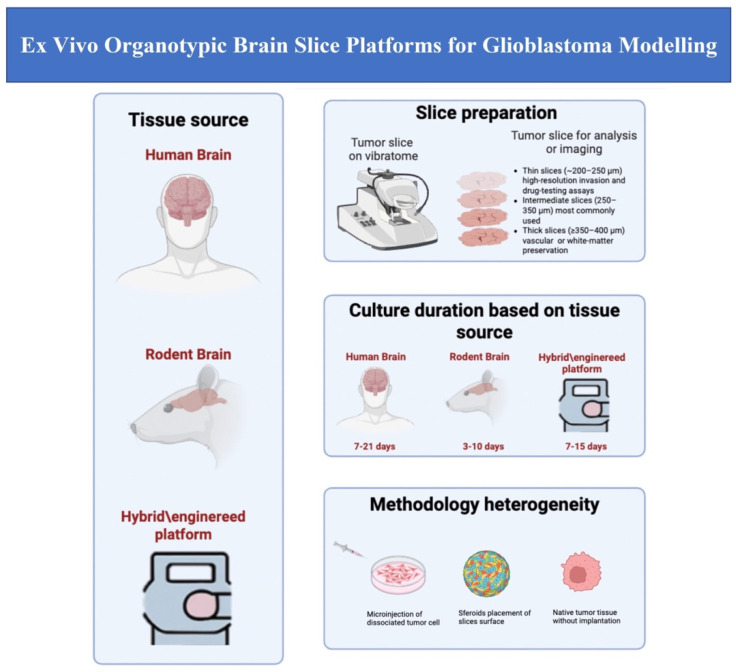
Overview of ex vivo organotypic brain slice platforms and their main applications in glioblastoma research.

The 26 included studies applied organotypic slice platforms across three major research domains: tumour invasion, TME interactions, and therapeutic testing. An overview of the main application domains and key methodological features of the included models is illustrated in Figure 2.

#### 3.4.1. Tumour Invasion and Migration

The majority of studies (*n* = 18; 69%) employed organotypic slices to evaluate GBM cell invasion. The most common measured outcomes to evaluate migration were radial invasion distance, time-lapse migration speed, trajectory mapping, perivascular tracking and ECM-associated motility patterns [26,29,33,40].

Rodent-based invasion models

The rodent slice model provided a highly controlled and reproducible platform to study the aforementioned outcomes.

Key contributions included the following:

- Gritsenko et al. identified peculiar perivascular and astrocyte-guided invasion preferred paths [26];
- Eisemann et al. instead perfectioned a model to quantify invasion distance [33];
- Decotret et al., in his paper, described an optimised 3D invasion assay (BraInZ) distinguishing invasion modalities [29];
- Linder et al. described how a solution with arsenic trioxide (ATO) together with gossypol could suppress GSC invasion [18].

Human-based invasion models

Human-derived slices enabled analysis of patient-specific invasion behaviour:

- Parker and his group in two different papers suggested marked intratumoural heterogeneity in single-cell migration trajectories and consequently in invasion patterns [21,37];
- Ravi et al. instead showed structural determinants of invasion microinjecting patient-derived cells [22];
- Liu et al. quantified how TNF-related apoptosis-inducing ligand (TRAIL)-induced apoptosis modifies residual invasive fronts [27];
- Merz et al. showed variable invasion depth and proliferation within the same GBM tissue [19].

Key insights across invasion studies

Across the studies included we reported several consistent findings present both in rodent and human platforms:

- GBM cells’ ability to invade the surrounding tissues is strongly determined by white-matter tracts, vasculature and astrocytic boundaries [30,39];
- Perivascular migration was recurrently documented and this was consistent with the documented GBM behaviour [22,26];
- Slice thickness and oxygen tension have been reported as extremely relevant in terms of invasion patterns [22].

#### 3.4.2. Tumour–Microenvironment Interactions

A smaller (*n* = 6; 20%) but highly informative subset of studies examined interactions between tumour cells and glial, vascular, or immune cell populations belonging to the TME surrounding the tumour itself.

Microglia–tumour crosstalk

- Heiland et al. described that GBM cells can drive reactive astrocytes to adopt immunosuppressive states driven by microglia–tumour signalling [39];
- Ghoochani et al. documented tumour-induced angiogenesis and microglial activation in its Vascular Organotypic Glioma Impact Model (VOGIM) model [31];
- Raju et al. in 2015 demonstrated long-term maintenance of microglia and endothelial cells in human GBM slices allowing more in depth TME analysis [34].

Immune cell dynamics

- Anderson and its group in 2024 designed a model of an ex vivo slice environment with integration of T-cell infiltration and migration describing interactions in real time [35].

Tumour–stromal and ECM interactions

- Ravin et al. demonstrated how primary GBM cells preferentially migrate following blood vessels and very interestingly how oxygen concentration dynamically modulate their speed and migration patterns [41];
- Nickl et al. reported that patient-derived slices retain native stromal, vascular, and immune system providing a physiological environment for modelling therapeutic responses [24].

Key TME findings

- Preservation of astrocytes, microglia, vasculature and ECM has been found essential for reproducing relevant GBM behaviour;
- Patient-derived slices uniquely retain immune and stromal components which are basically absent in organoids, spheroids or in 2D systems;
- Ex vivo TME dynamics often replicate in vivo patterns such as perivascular invasion and angiogenic remodelling.

#### 3.4.3. Therapeutic Testing

Studies testing therapeutic agents or modalities (*n* = 18; 70%) were highly heterogeneous, spanning from classical chemotherapies to targeted inhibitors, radiotherapy, metabolic modulators and microfluidic drug delivery approaches. Organotypic models showed high flexibility, allowing for the quantitative assessment of treatment response within a preserved microenvironment.

Chemotherapy and targeted agents

- Merz et al. described a model assessing patient-specific responses to temozolomide, X-rays and carbon ions [19];
- Minami and his team applied cisplatin, paclitaxel and tranilast showing distinct efficacy and toxicity profiles [20];
- Maier et al. reported instead how mGluR3 inhibition sensitises GBM cells to alkylating agents [38];
- Linder et al. applied on their model a mix of arsenic trioxide and gossypol reporting a synergistic killing of GSCs coupled to regrowth suppression [18];
- Nickl et al. showed how patient-derived GBM slices have heterogeneous responses to temozolomide, lomustine and targeted compounds [24];
- Xu et al. added to their slices the HDAC inhibitor vorinostat inducing histone hyperacetylation and anti-proliferative effects in human glioma slices [23].

Microfluidic and engineered platforms

- Ravi et al. used human cortical organotypic slices to test environmental and pharmacological perturbations, demonstrating preserved microenvironmental architecture suitable for assessing GBM cell responses [22];
- Mann et al. described a patient-tailored drug screening using a mixed-species engineered organotypic system [25].

Virotherapy

- Liu et al. delivered soluble TRAIL (TNF-related apoptosis-inducing ligand) induced dose-dependent apoptosis and reduced tumour mass in human glioma slices [27].

Key therapeutic insights

- Patient-derived slices can mirror clinical behaviour, supporting their translational value for predicting drug sensitivity and resistance to treatment [19,24,38];
- Tissue architecture, slice thickness and exposure conditions have been described as strong modulators of treatment efficacy, emphasising the importance of drug penetration and also of the local microenvironment [20,42];
- Organotypic platforms revealed resistance patterns linked to specific microenvironmental niche areas such as hypoxic regions, astrocytic boundaries and perivascular zones that are not detectable in conventional 2D cultures [22,39].

### 3.5. Summary of Findings

Across the 26 rigorously selected studies, organotypic brain slice cultures consistently demonstrated high translational relevance as intermediary models designed to serve as a natural bridge between classical in vitro systems and in vivo experiments (a synthesis of the principal methodological variables and research domains is provided in Table 2).

Key strengths identified

- Preservation of human cytoarchitecture, vasculature, ECM, and stromal populations allows modelling of tumour behaviour in a closer to physiology context;
- Faithful reproduction of GBM invasion patterns, notably perivascular, white-matter and astrocytic boundary modulated migration, documented both through rodent and human models;
- Robust TME-related insights, particularly microglia–tumour communication, were strongly and repeatedly confirmed across all types of models;
- Ability to evaluate drug responses in patient-specific environments, supporting potential future applications in functional precision oncology.

Major limitations and variability

- Substantial high methodological heterogeneity, including differences in slice thickness, media composition, oxygenation, tumour implantation methods and culture duration;
- Incomplete reporting across the majority of the studies regarding metrics viability, oxygen control, experimental blinding, and sample-size justification, therefore significantly reducing comparability across studies;
- High variability in perfusion and drug delivery conditions, especially between static cultures and engineered platforms affecting drug penetration and effect sizes;
- Species differences, particularly between rodent and human slices, affecting immune cell retention, vascular integrity and invasion phenotypes.

Summary of findings

From our review, we can summarise that organotypic brain slice models provide a robust, physiologically relevant platform to investigate GBM invasion, tumour–microenvironment interactions and therapeutic responses to treatment. In fact, despite methodological heterogeneity, the collective evidence supports their critical role within modern GBM research and their growing relevance in translational and precision oncology pipelines. Future progress will depend on methodological standardisation, including media formulation, oxygen regulation, viability reporting and precise quantitative invasion metrics allowing reproducibility and facilitating broader clinical adoption.

## 4. Discussion

A key point emerging from this systematic review is that the different organotypic brain slice culture models serve a complementary, rather than a hierarchical, role in GBM research. Rodent-derived slice cultures have historically been the most widely adopted platform due to their high reproducibility, technical robustness and accessibility, making them particularly suitable for mechanistic studies focused on tumour invasion, migration, and pathway evaluation. However, the obvious intrinsic species mismatch limits their capacity to fully recapitulate the complexity of the human tumour microenvironment.

In contrast, patient-derived organotypic brain slices preserve native human cytoarchitecture, tumour heterogeneity, and microenvironmental components, enabling the investigation of patient-specific invasion patterns and therapeutic responses with greater biological fidelity. These features make human-derived slices especially relevant for translational and precision oncology applications, despite reduced availability and increased inter-sample variability. Finally, hybrid and engineered platforms, including xenograft-derived slice cultures and microfluidic-integrated systems, further extend these approaches by combining preserved tumour architecture with improved control over perfusion, oxygenation, and drug exposure. Overall, these considerations indicate that model selection should be driven by the specific experimental objective, with rodent-based systems best suited for controlled mechanistic investigations and patient-derived or hybrid models preferred when translational relevance and functional drug testing are prioritised.

### 4.1. Invasion Biology

Organotypic slice cultures remain uniquely suited to study GBM invasion. Notably, human-derived slices reproduced patient-specific patterns of perivascular cell and white-matter migration [19,22,37], while rodent-based systems provided highly reproducible invasion assays, enabling quantification of radial distance, directionality and astrocyte-associated paths [26,29,33]. Together, these models demonstrate how microenvironmental architecture shapes GBM cells’ motility in ways that other in vitro models cannot reproduce due to their inherent limitations.

### 4.2. Therapeutic Testing and Translational Potential

Organotypic slice culture models consistently demonstrated the ability to reflect clinically relevant responses. For instance, human slices captured patient-specific sensitivity to temozolomide, radiotherapy and targeted agents [19,24] also supporting personalised drug screening approaches [25]. In addition, rodent models or mixed-species systems elucidated drug–response pathways, including mGluR3-mediated chemosensitisation [38], anti-proliferative effects of vorinostat [23] and synergistic activity of arsenic trioxide and gossypol against GSCs [18]. Finally, engineered or microfluidic systems addressed key challenges regarding drug penetration and perfusion [22,25], offering higher experimental control over exposure conditions. However, treatment efficacy remained strongly affected by several variables, including tissue architecture and microenvironmental gradients.

### 4.3. Methodological Variability and Challenges

Variations in areas such as slice thickness, media composition, oxygenation, tumour implantation methods and culture duration severely affected viability, invasion measurements and stromal preservation, further contributing to the considerable heterogeneity. Moreover, reporting quality was similarly inconsistent: viability controls were rarely systematic, oxygenation was largely undocumented and almost none of the studies reported blinding, randomisation or sample-size justification. This variability clearly limits cross-study comparability and represents a major barrier to broader translational applications.

## 5. Conclusions

The collective evidence extracted demonstrates that organotypic slice cultures are among the most physiologically relevant platforms for studying GBM invasion, TME interactions and therapeutic responses. Their principal strength lies in their capacity to retain native cytoarchitecture and immune–stromal elements, providing a level of biological fidelity that naturally serves as a bridge between in vitro and in vivo models. As engineered and microfluidic systems continue to advance, improvements in tissue perfusion, viability and drug delivery control are further enhancing the translational potential of slice-based models. Future progress will be based on methodological standardisation, in particular regarding media formulation, oxygen delivery regulation and other key experimental domains, to improve reproducibility and support integration into clinically oriented precision oncology workflows.

## Figures and Tables

**Table 2:** Classification and general characteristics of ex vivo organotypic brain slice models applied to glioblastoma research in the included studies.

Authors (Year)	Tissue Source	Slice Thickness (µm)	Tumour Modelling Approach	Culture Duration	Main Experimental Application
Xu (2011)	Human GBM surgical tissue	300	Native tumour tissue slice	3–5 days	Therapeutic response (drug testing)
Liu (2011)	Human GBM surgical tissue	300–350	Native tumour tissue slice	≤7 days	Therapeutic response (drug testing)
Natsume (2011)	Adult rodent brain	300	Cell implantation onto host slice	≤7 days	Invasion/migration (mechanistic)
Raju (2015)	Human GBM surgical tissue	300	Native tumour tissue slice	Up to 35 days	Methodological optimisation
Ghoochani (2016)	Adult rodent brain	350	Cell implantation onto host slice	8–10 days	Tumour–microenvironment interaction
Rinkenbaugh (2016)	Adult rodent brain	300	Cell implantation onto host slice	≤7 days	Invasion/migration (mechanistic)
Minami (2017)	Adult rodent brain	300	Native tumour tissue slice	≤7 days	Therapeutic response (drug testing)
Gritsenko (2017)	Adult rodent brain	300	Spheroid implantation onto host slice	5–7 days	Invasion/migration (mechanistic)
Parker (2017)	Human GBM surgical tissue	300–350	Native tumour tissue slice	≤15 days	Invasion/migration (quantitative)
Parker (2018)	Human GBM surgical tissue	300–350	Native tumour tissue slice	≤14 days	Invasion/migration (quantitative)
Eisemann (2018)	Adult rodent brain	300–350	Cell implantation onto host slice	≤7 days	Invasion/migration (quantitative)
Marques-Torrejon (2018)	Adult rodent brain	300	Microinjection of tumour cells	≤21 days	Tumour–microenvironment interaction
Merz (2013)	Human GBM surgical tissue	300–350	Native tumour tissue slice	≤21 days	Therapeutic response (radio/chemo)
Ravi (2019)	Human nontumoural cortical tissue	350	Microinjection of tumour cells	≤14 days	Invasion/migration (mechanistic)
Linder (2019)	Adult rodent brain	250–300	Spheroid implantation onto host slice	≤7 days	Therapeutic response (drug testing)
Heiland (2019)	Adult rodent brain	300	Cell implantation onto host slice	≤10 days	Tumour–microenvironment interaction
Sidorcenco (2020)	Human GBM surgical tissue + adult rodent brain	300	Tumour slice co-culture (tandem)	≤10 days	Invasion/migration (quantitative)
Hu (2020)	Human GBM surgical tissue	300–350	Native tumour tissue slice	≤7 days	Therapeutic response (drug testing)
Maier (2021)	Human nontumoural cortical tissue	300	Cell implantation onto host slice	≤10 days	Therapeutic response (radio/chemo)
Baeza-Kallée (2023)	Adult rodent brain	250	Spheroid implantation onto host slice	≤7 days	Therapeutic response (drug testing)
Nickl (2023)	Human GBM surgical tissue	350	Native tumour tissue slice	10–14 days	Therapeutic response (drug testing)
Decotret (2023)	Adult rodent brain	300	Spheroid implantation onto host slice	≤7 days	Invasion/migration (quantitative)
Mann (2023)	Human GBM surgical tissue + adult rodent brain	300–350	Tumour fragment co-culture	≤21 days	Therapeutic response (drug testing)
Sun (2023)	Adult rodent brain	300	Tumour fragment implantation	7–14 days	Therapeutic response (drug testing)
Ravin (2023)	Human nontumoural cortical tissue	350	Cell implantation onto host slice	≤14 days	Invasion/migration (mechanistic)
Anderson (2024)	Adult rodent brain	300	Cell co-culture onto host slice	≤7 days	Tumour–microenvironment interaction

**Table 3:** Methodological features, experimental applications and reported limitations of ex vivo organotypic brain slice models across the included studies.

Authors (Year)	Slice Preparation	Culture Configuration	Culture Medium	Experimental Intervention(s)	Outcome Measures	Main Application	Key Limitations
Xu (2011)	Vibratome	Submerged	Serum-containing	Chemotherapy testing	Viability/apoptosis	Drug screening	Short-term culture
Liu (2011)	Vibratome	Submerged	Serum-containing	Gene therapy	Viability/apoptosis	Drug screening	Short-term culture
Natsume (2011)	Vibratome	Air–liquid interface	Serum-containing	Targeted genetic modulation	Invasion/migration	Invasion modelling	Rodent host tissue
Raju (2015)	Manual explants	Submerged	Multiple media compared	None (model development)	Viability/apoptosis	Methodological development	Short-term culture
Ghoochani (2016)	Vibratome	Air–liquid interface	Serum-containing	Chemotherapy testing	Invasion/migration	Drug screening	Rodent host tissue; short-term culture
Rinkenbaugh (2016)	Vibratome	Air–liquid interface	Serum-containing	Targeted pathway inhibition	Imaging-based growth	Drug screening	Rodent host tissue; short-term culture
Minami (2017)	Vibratome	Air–liquid interface	Serum-free	Chemotherapy testing	Invasion/migration	Drug screening	Rodent host tissue
Gritsenko (2017)	Vibratome	Air–liquid interface	Serum-containing	None (model validation)	Invasion/migration	Invasion modelling	Rodent host tissue; short-term culture
Parker (2017)	Vibratome	Air–liquid interface	Neurobasal-based	None (model development)	Invasion/migration	Invasion modelling	Short-term culture
Parker (2018)	Vibratome	Air–liquid interface	Neurobasal-based	Targeted pathway inhibition	Invasion/migration	Invasion modelling	Short-term culture
Eisemann (2018)	Vibratome	Air–liquid interface	MEM-based	Targeted pathway inhibition	Invasion/migration	Invasion modelling	Rodent host tissue
Marques-Torrejon (2018)	Vibratome	Air–liquid interface	Serum-free	Chemotherapy testing	Viability/apoptosis	Tumour–microenvironment interactions	Rodent host tissue
Merz (2013)	Vibratome	Air–liquid interface	Serum-containing	Chemotherapy/Radiotherapy	Viability/apoptosis	Drug screening	Limited tissue availability
Ravi (2019)	Vibratome	Air–liquid interface	Neurobasal-based	None (model development)	Immune/TME readouts	Tumour–microenvironment interactions	Limited tissue availability
Linder (2019)	Vibratome	Air–liquid interface	Serum-free	Chemotherapy testing	Viability/apoptosis	Drug screening	Rodent host tissue
Heiland (2019)	Vibratome	Air–liquid interface	Serum-free	Immunomodulation	Immune/TME readouts	Tumour–immune interactions	Limited tissue availability
Sidorcenco (2020)	Vibratome	Tandem/hybrid culture	Serum-containing	Targeted pathway inhibition	Invasion/migration	Drug screening	Rodent host tissue
Hu (2020)	Vibratome	Air–liquid interface	Serum-containing	Targeted pathway inhibition	Imaging-based growth	Methodological development	Rodent host tissue
Maier (2021)	Vibratome	Air–liquid interface	Serum-containing	Chemotherapy testing	Viability/apoptosis	Drug screening	Limited tissue availability
Baeza-Kallée (2023)	Vibratome	Air–liquid interface	Serum-containing	Targeted pathway inhibition	Invasion/migration	Invasion modelling	Rodent host tissue
Nickl (2023)	Vibratome	Air–liquid interface	Serum-containing	TTFields	Viability/apoptosis	Precision oncology	Short-term culture
Decotret (2023)	Vibratome	Air–liquid interface	Serum-containing	None (model development)	Invasion/migration	Methodological development	Rodent host tissue
Mann (2023)	Vibratome	Air–liquid interface	Serum-containing	Chemotherapy testing	Imaging-based growth	Precision oncology	Rodent host tissue
Ravin (2023)	Tissue chopper	Air–liquid interface	Serum-containing	None (model development)	Invasion/migration	Invasion modelling	Limited tissue availability
Anderson (2024)	Vibratome	Submerged	Serum-containing	Targeted pathway inhibition	Invasion/migration	Invasion modelling	Rodent host tissue

## Data Availability

No new data were created or analysed in this study. Data sharing is not applicable to this article.

## Abbreviations

ATO	arsenic trioxide;
DMEM	Dulbecco’s modified Eagle’s medium;
ECM	extracellular matrix;
GBM	glioblastoma;
GSC	glioma stem cell;
HDAC	histone deacetylase;
LDH	lactate dehydrogenase;
PRISMA	Preferred Reporting Items for Systematic Reviews and Meta-Analyses;
ROBINS-I	Risk Of Bias In Non-randomised Studies;
SYRCLE	Systematic Review Centre for Laboratory animal Experimentation;
TME	tumour microenvironment;
TNF	tumour necrosis factor;
TRAIL	tumour necrosis factor-related apoptosis-inducing ligand;
TTFields	tumour-treating fields;
TUNEL	terminal deoxynucleotidyl transferase dUTP nick end labelling;
VOGIM	Versatile Organotypic Glioma Invasion Model.

## Appendix A

**Table A1:** Optimal vs. suboptimal methodological parameters for ex vivo organotypic brain slice models in glioblastoma research. This table summarises key methodological parameters reported across the included studies, highlighting conditions associated with optimal tissue viability, preservation of microenvironmental features, and experimental readouts, as well as parameters linked to suboptimal performance. The classification is based on qualitative synthesis of the reviewed literature and reflects commonly reported experimental practices rather than formal quantitative thresholds.

Parameter	Optimal Range/Condition	Suboptimal Condition	Impact on Functional Outcomes
Slice thickness	250–350 µm	<200 µm or >400 µm	Excessively thin slices compromise cytoarchitecture, while overly thick slices develop hypoxia and reduced viability, affecting invasion and drug–response readouts
Tissue source	Patient-derived GBM tissue	Long-term established cell lines only	Reduced tumour heterogeneity and loss of patient-specific invasion and treatment response patterns
Culture duration	Short- to mid-term (3–14 days)	Prolonged culture without optimisation	Progressive loss of tissue viability and altered microenvironmental signalling
Oxygenation strategy	Interface culture or controlled perfusion	Static submerged culture	Impaired oxygen diffusion, central necrosis, and altered invasion dynamics
Culture medium	Serum-free, neurobasal-based formulations	Serum-containing media	Artificial differentiation and non-physiological tumour behaviour
Tumour implantation strategy	Native tumour slices or controlled microinjection	Irregular or poorly controlled seeding	Reduced reproducibility and increased variability of invasion assays
Functional readouts	Multimodal assessment (imaging, viability, molecular markers)	Single endpoint analysis only	Incomplete or misleading interpretation of tumour behaviour and therapeutic response

**Supplementary Methods A1—PubMed search strategy**

The PubMed database was searched using the following Boolean string:

**(glioblastoma OR GBM)** AND (organotypic brain slice OR brain slice culture OR ex vivo brain slice* OR organotypic slice*)** AND **(invasion OR tumour microenvironment OR tumor microenvironment OR therapeutic response OR drug response)**

Filters applied: English language; publication date from 1 January 2010 to 31 July 2025.
